# Alternative Food Networks in Latin America—exploring PGS (Participatory Guarantee Systems) markets and their consumers: a cross-country comparison

**DOI:** 10.1007/s10460-022-10347-w

**Published:** 2022-08-25

**Authors:** Sonja Kaufmann, Nikolaus Hruschka, Luis Vildozo, Christian R. Vogl

**Affiliations:** Department of Sustainable Agricultural Systems, Institute of Organic Farming, University of Natural Resources and Life Sciences, Vienna, Gregor-Mendel-Str. 33, 1180 Vienna, Austria

**Keywords:** Alternative Food Networks, Participatory Guarantee Systems, Farmers’ market, Consumers, Organic certification, Latin America

## Abstract

Alternative food networks (AFN) are argued to provide platforms to re-socialize and re-spacealize food, establish and contribute to democratic participation in local food chains, and foster producer–consumer relations and trust. As one of the most recent examples of AFN, Participatory Guarantee Systems (PGS) have gained notable traction in attempting to redefine consumer-producer relations in the organic value chain. The participation of stakeholders, such as consumers, has been a key element theoretically differentiating PGS from other organic verification systems. While research on farmer participation in PGS is attracting interest, consumer participation is still widely overlooked. Using a mixed methods approach, this paper describes five PGS markets in Mexico, Chile and Bolivia. A survey was conducted with consumers in the PGS markets to explore their awareness of the PGS, how consumers participate in the PGS, and their level of trust in the respective PGS and its certified products. Results showed a low level of awareness of PGS among market consumers, few participation possibilities, and minimal consumer participation overall. Nevertheless, trust in organic quality was generally high. Consumers primarily relied on the direct relationship with producers and the PGS market itself as sources of trust. These results provide novel insight into PGS consumer-market interactions, and contribute to discussions concerning social embeddedness, awareness and participation within AFN.

## Introduction

Public policies promoting organic agriculture, supporting family farming, and addressing food security have made their mark in Latin America in recent decades (Flores 2019). While the increase in organically managed land is noteworthy (Lernoud et al. 2019), with a share of 0.8% on global organic retail sales and a per capita consumption of 1.50€ in 2019 (Willer et al. 2021), the organic market in Latin American countries still strongly represents a niche. Price premiums for organic products and low consumer awareness have been described as barrier for the development of organic markets in several Latin American countries (Agence BIO 2021), where the share of consumers’ expenditure on food is still relatively high^1^ when compared to key organic markets such as Germany and the US (Global Organic Trade Guide 2022a, b, c, d, e, f). However, as a region historically characterized by organic export-orientated production (Berdegué and Fuentealba 2011) the rise in domestic demand for organic produce in Latin America is of growing interest (Berdegué and Fuentealba 2011; Flores 2019). The development of the domestic Latin American organic market, strongly supported by *Alternative Food Networks (AFN),* community-based initiatives, and civil society organizations, has been described as an important business opportunity for smallholder farmers and a key factor in promoting organic farming and increasing the sustainability of food systems (Flores 2019).

As part of this development, *Participatory Guarantee Systems (PGS)* have become an increasingly popular approach for certifying the organic produce of smallholder farmers producing for domestic markets. PGS are local conformity assessment systems that are designed to guarantee organic integrity through the participation and ownership of all stakeholders (Bouagnimbeck 2014; IFOAM 2008). PGS stakeholders, such as producers, NGOs and consumers, are presumed to participate actively in the organic verification process, decision-making and organizational activities, thus establishing trust, promoting participation, conveying transparency, creating social networks, and encouraging shared learning processes and knowledge exchange (IFOAM 2008, 2019). As such, PGS are attracting increasing attention and becoming popular as an alternative to third-party certification (TPC). PGS are intended to make organic certification more suitable for smallholder farmers (Bouagnimbeck 2014) and often aim to endorse a more holistic ideology of organic farming (Nelson et al. 2010)—basing their activities on the principles of agroecology (Rover et al. 2017) – or counteract conventional market dynamics (Nelson et al. 2010).

While organic quality assurance is at the core of PGS, many PGS initiatives have been found to engage in collective commercialization activities (Kaufmann et al. 2020). Producers participate in (Hruschka et al. 2022) or organize their own (Bellante 2017; Binder and Vogl 2018; Rover et al. 2017; Sacchi et al. 2015) farmers’ markets at which consumers are able to buy PGS-certified organic produce. Consequently, PGS can be understood as AFN that are governed by self-established conventions for participatory conformity assessment and organic production, and promote socially-embedded, short food supply chains, thereby dis-embedding themselves from the conventional food industry and the conventionalization of organic farming (Bellante 2017; Brunori et al. 2012; DuPuis and Goodman 2005; Maye and Kirwan 2010). Thus, PGS provide not only an alternative to TPC, but also a different approach to consumer-producer relations and access to organic food.

In PGS and AFN, the intention is for consumers to be actively involved in starting and operating the initiatives (Bouagnimbeck 2014; IFOAM 2019; Renting et al. 2012). PGS activities potentially allowing for consumer involvement include farm visits as part of the organic guarantee system, workshops and training courses, diverse events or organizational activities (Kaufmann et al. 2020). Unfortunately, there has been a lack of a more in-depth examination of consumers and their participation in PGS activities in scholarly literature (Kaufmann et al. 2020; van Truong et al. 2022), as analyzed and summarized more thoroughly by Kaufmann et al. (2020). Although several authors found consumers to be part of initial PGS development and implementation (Niederle et al. 2020; López Cifuentes et al. 2018), quantifiable empiric evidence on consumer participation is scarce and studies referring to consumer participation often refer to the PGS design rather than its observed implementation (Kaufmann et al. 2020). Most recent studies explored the role and involvement of consumers in ethical purchasing groups engaged with PGS producers (Sacchi 2018; Sacchi et al. 2022), the effects of system design and direct participation on stakeholder trust (Thamchaisophis 2021), and consumer trust in different certification schemes, including PGS (van Truong et al. 2022). Some of these studies found active consumer participation, e.g. in providing consulting service for the PGS certification of producers supplying their purchasing group (Sacchi 2018). Consumer participation in PGS is widely recommended by IFOAM (Bouagnimbeck 2014; IFOAM 2019). It is a key element differentiating PGS from other organic certification systems (Bouagnimbeck 2014; May 2008) and has been noted by several scholars as crucial to guaranteeing the success of PGS initiatives (Clark and Martínez 2016; Home et al. 2017) by boosting stakeholder trust, knowledge exchange, empowerment and the sharing of responsibility for the conformity assessment system (IFOAM 2018, 2019). Active involvement of consumers has been found to promote trust-building (Thamchaisophis 2021) and empowerment in terms of community decision-making (Sacchi et al. 2022). Nevertheless, previous PGS research also indicates that motivating consumers to be participating stakeholders in PGS activities is something of a challenge (López Cifuentes et al. 2018; Nelson et al. 2016; Bara et al. 2017).

Apart from their active involvement in PGS activities, consumers also have a key role to play in the purchase of PGS products. A stable market for PGS products is needed if PGS producers and initiatives are to maintain their activities. In this context, consumer awareness of and trust in PGS certification are key (Batte et al. 2007; Darby and Karni 1973; Janssen and Hamm 2012; Kriege-Steffen et al. 2010; Roitner-Schobesberger et al. 2008) and play a crucial role in the sustainable functioning of PGS and their markets and their ongoing promotion of domestic organic sectors in Latin America (Giovannucci and Ponte 2005; Higgins et al. 2008; Maye and Kirwan 2010).

This paper aims to contribute to closing the research gap concerning consumers in PGS by exploring the role of consumers in five Latin American PGS and their markets. To learn from and contribute to a broader discourse on AFN and farmers’ markets in Latin America, this study is positioned within the discourse on AFN and selected concepts of AFN theory. The paper starts by outlining key concepts in AFN literature, including social embeddedness and farmers’ markets, and organic consumer behavior. Secondly, it describes the research approach, methods and case studies conducted in Mexico, Chile and Bolivia. Finally, findings on consumer awareness, participation and trust are presented and discussed.

### Alternative Food Networks (AFN)

Research on the impact of AFN on consumer-producer relations has attracted considerable attention in scholarly literature (Goodman 2004; Michel-Villarreal et al. 2019; Renting et al. 2003; Seyfang 2006). Encompassing a wide range of social and economic backgrounds, AFN emerge from political, cultural and historical processes to challenge conventional, global food supply chains by establishing localized, high-quality food supply chains (Jarosz 2008; Murdoch et al. 2000; Renting et al. 2003). Within the increasingly blurred concepts of alternative and conventional food systems (Barbera et al. 2020; Sonnino 2007; Tregear 2011), the difficulty in pinpointing a specific definition of AFN (Jarosz 2008; Renting et al. 2003; Tregear 2011; Edwards 2019) without conflating inherent structures, characteristics and outcomes (Tregear 2011) is ever more present. By being dis-embedded from the conventional food industry (Brunori et al. 2012; DuPuis and Goodman 2005), it is argued that AFN provide platforms to re-socialize and re-spacealize food (Renting et al. 2003), establish and contribute to democratic participation in local food chains (Hinrichs 2003), and build stakeholder trust (Martindale 2020). Undeniably, issues regarding exclusionary characteristics of AFN and its system boundaries impeding participation have been raised (Edwards 2019; Kato and McKinney 2015; Slocum 2007). Although strategies have been proposed to promote participation of a broader spectrum of consumers (Allen 2010), authors have indicated elitism and the participation of a middle-class strata to be central characteristics of AFN consumers (Kato and McKinney 2015; Slocum 2007). Moreover, issues regarding the extent to which AFN offer cultural and societal inclusivity outside of a predominantly white audience have been raised (Kato and McKinney 2015). Yet the emergence of AFN, in which consumer inclusion is fostered and participatory forms of self-management and self-organization are paramount, allegedly has led to the establishment and promotion of new forms of consumer-producer behavior and relations (Barbera et al. 2020; Renting et al. 2003, 2012; Winter 2003). Undeniably, producer–consumer interactions have become a recurrent point of interest in AFN research (Barbera et al. 2020; Dubois 2018) and it is increasingly relevant to have a better understanding of proactive citizen-consumers (Renting et al. 2012; Soper 2007).

The understanding of *consumer behavior* has shifted from a classical and neoclassical economics perspective, in which consumers are treated as self-interested, rational economic actors (Feagan and Morris 2009; Granovetter 1985; Hinrichs 2000), towards exploring how consumers’ economic behavior is embedded in a complex mesh of social relations (Granovetter 1985; Polanyi et al. 2001). Granovetter (1985) highlights the importance of social and interpersonal relations for generating trust and discouraging malfeasance in economic transactions, thereby introducing *social embeddedness* as an additional approach to marketness and instrumentalism, i.e. the price signal and prioritization of economic factors over other motivations as drivers of consumer behavior in economic activities (Feagan and Morris 2009; Hinrichs 2000). In the last few decades, embeddedness research has received widespread acknowledgement within AFN research (Feagan and Morris 2009; Kirwan 2004; Sage 2003; Sonnino and Marsden 2006). Even though partially contested (Sayer 2001), specifically, as social embeddedness does not directly equate to equal power distribution or absence of intolerance amongst AFN participants (Hinrichs 2003), the importance of considering social embeddedness, specifically social connection, trust and reciprocity to analyze and uncover social structures within economic activities in AFN cannot be overlooked (Barbera et al. 2020; Sonnino 2007).

Short food supply chains have been highlighted as another key concept in AFN theory (Maye and Kirwan 2010) as direct marketing channels that are “‘embedded’ with value-laden information concerning the mode of production, provenance, and distinctive quality assets of the product” (Maye and Kirwan 2010, p.3). Recent empirical AFN studies have focused primarily on consumer purchasing groups (Sacchi 2018; Sacchi et al. 2022), Community Supported Agriculture (CSA) initiatives and *farmers’ markets* (Michel-Villarreal et al. 2019). Although literature indicates that a clear demarcation of how AFN differentiate from and provide an “alternative” to mainstream capitalist market systems (Edwards 2019; Jarosz 2008) is lacking, farmers’ markets have been argued to be places of socialization (Dodds et al. 2014; Hinrichs 2000), material and non-material exchange (Carson et al. 2016; Holloway et al. 2007; Kirwan 2004), and characterized by face-to-face producer–consumer interactions that establish trust (Thorsøe and Kjeldsen 2016). Farmers’ markets are therefore viewed as being socially embedded (Hinrichs 2000), entrenching attributes and values such as knowledge about production processes, locally-produced, organic, high-quality food, and trust-based relationships that accompany and/or go beyond the relevance of price (Feagan and Morris 2009 cit. Holloway and Kneafsy 2000, O’Hara and Stagl 2001, Hunt 2007).

Although the social, economic and ecological benefits of farmers’ markets have partially been explored, recent research has primarily focused on profiling consumers and analyzing markets’ economic impact (Figueroa-Rodríguez et al. 2019), and only a slowly growing number of studies are contributing to the understanding of social embeddedness and non-economic aspects of farmers’ markets (Carson et al. 2016; Kirwan 2004; Klimek et al. 2018). Farmers’ markets are said to facilitate the exchange of information and reduce information asymmetries, thereby contributing to consumer awareness, learning about sustainable food systems (Forssell and Lankoski 2015) and consumer behavior favoring organic foods (Carson et al. 2016).

Shared producer–consumer knowledge (Sacchi 2018) and the provision of sufficient product information have been formulated as one concept for the generation of consumer *trust* in AFN (Thorsøe and Kjeldsen 2016). Trust is not only a prerequisite of AFN consumption (Giampietri et al. 2018; Martindale 2020) and AFN social relations (Sacchi et al. 2022). Through consumer trust, AFN could establish coherency, facilitate co-operation (Thorsøe and Kjeldsen 2016), manage food network knowledge asymmetries, and mobilize consumers to participate in AFN activities (Jarosz 2008; Martindale 2020).

Sufficient information flows towards consumers have also been highlighted as a key factor in supporting the development of consumer trust in organic products (Kriege-Steffen et al. 2010). When organic products are concerned, this is particularly important as organic product characteristics are credence attributes that consumers cannot determine for themselves prior to purchasing a product (Darby and Karni 1973; Jahn et al. 2005). Hence, for consumers to purchase organic food products it is crucial that they are adequately educated on the matter (Kriege-Steffen et al. 2010; Roitner-Schobesberger et al. 2008). Direct producer–consumer interactions could give farmers’ markets a distinct opportunity to foster this information exchange (Carson et al. 2016). Beyond direct communication, organic product quality is guaranteed and signaled to consumers by means of organic certification schemes, such as PGS, and corresponding labels (Janssen and Hamm 2012). Consumer awareness of these schemes and labels is key to their success (Janssen and Hamm 2012) and can further positively affect the probability of consumers being willing to pay organic price premiums and hence purchase organic foods (Batte et al. 2007; Janssen and Hamm 2012).

While research has been conducted on farmers’ markets and consumers primarily in North America and western Europe (Figueroa-Rodríguez et al. 2019; Yiridoe et al. 2005), where farmers’ markets have been closely associated with the development of the organic sector (Sahota 2020), empirical studies on organic farmers’ markets and their consumers in Latin America remain rare (Michel-Villarreal et al. 2019). Similarly, the consumer perspective has continuously been disregarded in AFN research (Goodman 2004; Michel-Villarreal et al. 2019; Tregear 2011) and barely addressed in PGS literature (Kaufmann et al. 2020) with the exception of a few scholars (Sacchi 2018; Sacchi et al. 2022; Giampietri et al. 2018).

## Research aim and approach

To contribute to closing this research gap, this paper uses empirical evidence to explore the role of consumers in five PGS case study markets (CSM) in Latin America. For this purpose, the study assessed consumers’ awareness of the PGS, their participation in PGS activities and their trust in PGS products. This ultimately allows suggestions to be made for the further development of PGS, farmers’ markets and consumer participation.

A mixed methods approach (Bernard 2006) was adopted with regard to PGS initiatives and their respective farmers’ markets in Mexico by the first author, Chile by the second author and Bolivia by the third author. Semi-structured interviews, document analysis and participant observations were used to depict how the initiatives commercialize products and communicate their organic quality, and how they approach consumer participation. Consumer surveys were conducted by the first and second author in Mexico and Chile respectively. The Bolivian consumer surveys were conducted by the third author and supported by a local student. The consumer surveys explored consumers’ PGS awareness, their level of subjective trust in PGS products, and their participation in PGS activities. To boost the insights acquired from these data, statistical tests were computed to compare samples across the three study countries.

The results section is organized into three sub-sections corresponding to the three elements of the role of consumers in PGS explored in this paper. The first two sub-sections are structured as follows: first, the CSMs’ approach to consumer awareness and participation is depicted by describing (i) how they communicate the PGS concept and the products’ PGS certification status to consumers, and (ii) opportunities for direct participation at the market sites and in the PGS, and second, based on consumer survey data, the study explored (i) consumer awareness of the PGS concept and the PGS initiatives at the markets, and (ii) their willingness to take up these opportunities to participate. The third section explores consumer trust in the organic product quality based on consumer survey data. In each section, findings are discussed in the light of literature on PGS, AFN, farmers’ markets, and organic consumer behavior. Finally, the paper concludes with suggestions for the further development of CSMs and PGS.

## Materials and methods

The data presented in this paper are based on two studies. A first study was conducted in Mexico between September 2015 and March 2016. Following the insights gained in Mexico, a second study was planned and implemented by the authors in Chile (June 2019 – November 2019) and Bolivia (June 2019 – October 2019) as soon as additional funding was secured and after joint development, pretest, adaptation and calibration of wording in inquiry for all data collection instruments. The approach was the same for the second study in Bolivia and Chile as for the first study in Mexico. Data collection tools from Mexico were refined to add additional, more specific aspects of consumer awareness and participation in PGS. The results presented in this study portray the answers given to equivalent questions in the consumer surveys across all five CSMs during two separate data collection timeframes. In Mexico, data were collected in three local organic PGS markets (Mexican-CSM): Chapingo organic market (*Tianguis Orgánico Chapingo*), the alternative market of Tlaxcala (*Tianguis Alternativo Tlaxcala*), and the alternative market of Oaxaca “El Pochote Xochimilco” (*Tianguis Alternativo El Pochote Xochimilco*). In Chile and Bolivia, the *Ecoferia de la Reina* case study market (Chilean-CSM) in Santiago and the *ECO Feria* case study market (Bolivian-CSM) in Cochabamba were selected respectively.

### Data collection

Data were collected in four phases in each country (Table 1). In Phase 1, interviews were conducted with key actors, PGS and CSM representatives. The consumer survey was pre-tested with CSM consumers in Phase 2, and conducted in person with 201 consumers in Phase 3. Consumers were selected by convenience sampling on market days (Bernard 2006). Participant observation on market days and at PGS meetings (Bernard 2006) and informal interviews with PGS representatives and other key informants complemented the data collection throughout the study (Phase 4). Key documents provided by the PGS and CSMs were collected to supplement the data.

**Table 1:** Data collection overview of the Mexican case study markets (Mexican-CSM), the Chilean case study market (Chilean-CSM), and the Bolivian case study market (Bolivian-CSM) (n = number; data collection timeframe: *09.2015–03.2016; **06.2019–11.2019; ***06.2019–10.2019)

				Mexican-CSM*	Chilean-CSM**	Bolivian-CSM***
				(a) Chapingo (b) Tlaxcala (c) Oaxaca	(d) Ecoferia de la Reina	(e) ECO Feria Cochabamba
Phase	Method	Sampling strategy	Population	n	n	n
Phase 1	Semi-structured interviews	Purposive	PGS representatives, key informants	7 [1(a), 3(b),2(c), 1 (other)]	8 [4(d), 4 (other)]	2 [1(e), 1 (other)]
Phase 2	Consumer survey pre-tests	Convenience	Consumers	8 (a)	23 (d)	9 (e)
Phase 3	Consumer surveys	Convenience	Consumers	61 [21(a), 19(b), 21(c)]	82 (d)	58 (e)
Phase 4	Participant observation	Purposive, convenience	Market days, project meetings related to PGS	8	1	2
	Informal interviews	Purposive	PGS representatives, key informants	4	–	–

### Data analysis

Qualitative survey data regarding reasons for trust were coded inductively using first cycle descriptive coding and establishing 11 categories (Saldaña 2013) in Microsoft Excel (Microsoft 2016). Semi-structured interviews, informal interviews and observational protocols were deductively and inductively coded to address the CSMs context as well as the CSMs approaches on product labeling, consumer information and participation opportunities (Saldaña 2013). When citing from interviews, protocols and documents, the following syntax is used throughout the paper: “Country/material/number”, with country abbreviated by B (Bolivia), C (Chile) and M (Mexico), and material abbreviated by I (Interview), PO (Observation) and D (Document), followed by a consecutive number for each material item and country. Appendix A1 gives an anonymized overview and additional details of the materials used.

Quantitative analysis was conducted using IBM SPSS Statistics (Version 24). Contingency tables, Chi-square (χ^2^) test, Fisher’s exact test, Mann–Whitney U test, Kruskal–Wallis H test and Wilcoxon Signed-Rank test were applied to compare the sub-samples for the three study countries. Exact nonparametric tests were used for small and uneven sample sizes. To test for differences in consumer trust between the study countries using Fisher’s exact test, each of the three categories at the lower and upper ends of the six-level ordinal scale were combined to reduce the scale to three categories. If exact p values could not be computed due to the sample size, Monte Carlo p values were used. P values (p, exact p, Monte Carlo p) are labeled accordingly (Bühl 2016). For better readability of the paper, the results section depicts general descriptive statistics, while the test statistics for significance tests between countries and more detailed descriptive statistics are given in Appendices A2-A5.

### PGS case study markets

#### Mexican-CSM

The Mexican case study markets (Mexican-CSM) were founded in 2003 (Chapingo) (MPO1), 2005 (Tlaxcala) (MD2) and 2010 (Oaxaca) (MI9), and started implementing a PGS in their respective opening year (MPO1; MI3; MI5). The engagement of non-producing actors (i.e. consumers, academics, artists) played a key role in the initial development phase of Mexican-CSM (MI3; MI5; MD1). As is typical for Mexico, the PGS in Mexican-CSM served as an organic guarantee system for products sold at the respective market. Consequently, all CSM members who sold food products at the market were subject to the established PGS, and all producers and processors certified by the PGS also sold their products at the market. At the time of data collection, Mexican-CSM comprised 24 (Chapingo), 19 (Tlaxcala) and 37 (Oaxaca) stands selling food products or prepared meals that were subject to the PGS (MPO3; MPO4; MPO5). Mexican-CSM in Chapingo and Tlaxcala took place once a week, while the Mexican-CSM in Oaxaca had two market days per week.

#### Chilean-CSM

The Tierra Viva A.G. PGS (Tierra Viva), founded in 1993, did not run its own market (CI2). Following the closure of the Tierra Viva market due to low demand and an unsuccessful attempt by consumers to open a consumer cooperative (CI3), Tierra Viva producers are free to sell their produce in any markets and/or stores (CI2). The *Ecoferia de la Reina* (Chilean-CSM), an organic market located in Santiago, has become a central sales venue for many Tierra Viva producers (CI2). Chilean-CSM was founded in 2013 (CI3). At the time of data collection, Chilean-CSM operated twice a week and had between 28 and 30 stands, encompassing artisans, small retailers, one food stand and producers, 13 of whom sold certified organic products (five certified by Tierra Viva, one by PGS Orgánicos de Aconcagua, and seven by TPC bodies).

#### Bolivian-CSM

The ECO Feria PGS was founded in Cochabamba in 2003 with the assistance of the AGRECOL Andes Foundation (BI1), a non-profit organization. ECO Feria was granted PGS status in 2012 (BI1) and is part of the ECO Feria Association (BI2). The ECO Feria Association had 30 members, of whom 13 were certified organic and were active in the weekly *ECO Feria Cochabamba* market (Bolivian-CSM). In addition to producers certified by the ECO Feria PGS, one producer certified by the Agroecovit PGS and two producers certified by a TPC body sold at Bolivian-CSM. Artisans, small retailers and food stands were also present at Bolivian-CSM. The AGRECOL Andes foundation supported the ECO Feria with financial contributions, logistical support and training (BI1).

### CSM consumer survey participants

The CSM consumers surveyed were predominantly female, with an arithmetic mean age of 43 years. Seventy-eight percent of consumers had a university education. Market consumers travelled on average 22 min to the marketplaces of CSM (Appendix A2). According to key informants at the Mexican-CSM (Chapingo, Tlaxcala) and the Chilean-CSM, consumers were characterized by higher purchasing power (MI2, MI5, CI1, CI2). Due to issues related to data reliability, data resulting from a survey question regarding consumers’ household income were excluded from the analysis.

The arithmetic mean of consumer market attendance was three years. Almost two thirds of consumers had been going to the market for more than one year, and one fifth for more than five years (Appendix A3). Consumers at Bolivian-CSM had been attending the market for significantly less time than consumers at Chilean-CSM and Mexican-CSM. At Chilean-CSM and Mexican-CSM, more than one third had been attending the market for more than four years, while at Bolivian-CSM only 14% had done so. Only 43% of the respondents at Bolivian-CSM had been attending the market for more than one year (Appendix A3, Appendix A4).

Regarding consumers’ frequency of market attendance, the arithmetic mean was 35 visits per year (Appendix A5). The large majority of consumers (80.4%) visited their respective CSM more than once a month, over half visited the market more than twice a month, and slightly more than 40% approximately once a week. At Mexican-CSM and Chilean-CSM, almost half of the surveyed consumers visited the market weekly, while at Bolivian-CSM fewer than one third did so. Thus, consumer survey respondents at Bolivian-CSM attended the market significantly less frequently than those at Chilean-CSM (Appendix A4, Appendix A5).

## Results and discussion

### PGS consumers in the dark?

#### CSMs’ approach to creating consumer PGS awareness

The five CSMs took different approaches to product labeling and consumer information.

The three *Mexican*-CSM distinguished between different certification categories, including products in transition. The organic certification status of market products was communicated to consumers by displaying different colored table sheets (Chapingo; MI2; MPO1) or confirmation letters issued by the certification committee (Tlaxcala; MI3; MPO2; MI4; MI1) on the market stands. Product labels were not used as tools to display the certification status of PGS-certified products, and at Mexican-CSM in Oaxaca the PGS certification status was not visually signaled to consumers at all (MI5). To ensure that the food products sold at the market were exclusively certified by the PGS, in the Mexican-CSM in Tlaxcala members had established a supervising committee to check the type of products sold on market days and whether the product prices and PGS confirmation letter were being exhibited correctly, for example (MI3). At the Mexican-CSM in Chapingo and Oaxaca, no such system was observed (MPO3; MPO5). To provide consumers with information about the PGS and organic products, the Mexican-CSM in Chapingo had one information stand at the entrance to the market building, and in the forecourt of the market building a banner displayed a brief explanation of PGS (MPO3).

Similar to the Mexican-CSM, at the *Chilean*-CSM only certified organic food products were sold, but products were either certified by a PGS or by a third-party certification body. Initially non-certified and certified food products were sold in parallel (CI6), but for greater clarity and to avoid confusion and unfair advantages for non-organically certified producers, Chilean-CSM subsequently only allowed the sale of certified organic produce (CI6). Producers with products in transition were not allowed to sell their products (CI6). To communicate the certification status of a product to consumers, the certification body was displayed on the market stands’ name board and the indication “org” on most of the food product price signs highlighted the organic quality of the product. Some products also had a sticker of the local competent authority. Tierra Viva-processed products were labeled with the proprietary PGS label. Chilean-CSM also implemented an internal control system consisting of a market supervisor employed by Chilean-CSM regularly checking the organic certificates on market days (CI5). During these reviews, the organic certification validity was verified and a traceability check conducted, with the products sold cross-referenced with products listed on the organic certificate (CI5). Two farm inspections were also carried out by the market supervisor annually to verify the production and storage facilities (CI5). In the event of doubt, the market supervisor took samples on market days to be tested for prohibited substances (CI6). At Chilean-CSM, an information stand provided information for consumers.

At *Bolivian*-CSM the certification status of each product was defined by color stickers distinguishing between different categories and including products in transition and “made with organic ingredients” (BPO1). Organic certificates and the logos of ECO Feria and AGROECOL Andes were displayed at the stands on market days, but this approach was not adopted by all vendors. Several producers sold their produce at Bolivian-CSM as organic but were not able to show any certification.

The correct display of organic certificates and logos was checked once on each market day by the PGS representative. To provide consumers with information, market stands distributed promotional and informative material on PGS products prepared by the competent promotion authority.

#### Consumer awareness of PGS

With the exception of the Mexican-CSM in Oaxaca, all CSMs communicated the PGS-certified organic product quality to their customers. However, only a minority of consumers surveyed was *aware of PGS* (Table 2). While at Mexican-CSM this figure was close to one quarter, at Bolivian-CSM fewer than five percent of consumers had heard about PGS. PGS awareness among consumers at Bolivian-CSM was significantly lower than at Mexican-CSM and Chilean-CSM (Appendix A4). This dissimilarity could be related to different communication strategies and differences in consumer market attendance (Appendix A3), although statistical analysis did not substantiate this relation. Since Chilean-CSM and Bolivian-CSM sold products that were not certified by the PGS, consumers at Chilean-CSM and Bolivian-CSM were also asked whether they had heard of Tierra Viva and ECO Feria PGS respectively. In both cases, consumer awareness of the PGS initiative was greater than for PGS in general. Thus, awareness about a specific initiative does not automatically translate to PGS awareness, as consumers may not have identified Tierra Viva and ECO Feria as initiatives applying a PGS. Again, awareness was significantly lower for consumers at Bolivian-CSM than at Chilean-CSM (Table 2, Appendix A4).

**Table 2:** Consumer awareness of PGS in general and the respective PGS initiative at the Mexican case study markets (Mexican-CSM), the Chilean case study market (Chilean-CSM) and the Bolivian case study market (Bolivian-CSM) (n = number; n.a. = question not asked)

Variable	Total sample	Mexican-CSM Chapingo / Tlaxcala / Oaxaca [n = 61 (21/19/21), 100% = n)]	Chilean-CSM Ecoferia de la Reina (n = 82, 100% = n)	Bolivian-CSM ECO Feria Cochabamba (n = 58, 100% = 53)
Heard about PGS in general [yes] (n = 201, 100% = 196)	14.8%	24.6%	14.6%	3.8%
Heard about the PGS initiative [yes] (n = 140, 100% = n)	30.7%	n.a	39%	19%

Similarly, Bara et al. (2017) and Sacchi et al. (2015) have indicated low consumer awareness of PGS. Binder and Vogl (2018) even excluded consumers entirely from their study as they were unaware of PGS. However, the present results are not specific to PGS markets since other organic markets and AFN studies have also indicated a lack of consumer awareness of organic certification systems and standards (Giovannucci and Ponte 2005; Higgins et al. 2008; Janssen and Hamm 2012; Roitner-Schobesberger et al. 2008; Zagata and Lostak 2012). An information deficit concerning assurance procedures and certification bodies (Eden et al. 2008) and difficulties in adequately estimating the effort involved in complying with the standards corresponding to product labels are not uncommon (Hoogland et al. 2007). These findings were also reflected in the studied CSMs.

Only through awareness and understanding are consumers able to rationally valorize the credence values attributed to organically-certified products (Darby and Karni 1973; Zagata and Lostak 2012), and can organic labels become the tools they were designed to be (Smed et al. 2013; Testa et al. 2015). It is therefore argued in the literature that consumer awareness is key to the success of organic quality assurance systems and can be positively associated with consumers’ willingness to pay price premiums for organic products (Batte et al. 2007; Janssen and Hamm 2012), thus fostering consumer demand, which is a crucial factor for the success and growth of AFN (Higgins et al. 2008). Furthermore, awareness is the first step to establishing knowledge networks and trust relations in AFN (DuPuis and Goodman 2005).

### PGS consumer participation – an idealistic notion?

#### Participation opportunities in PGS and at CSMs

In the five CSMs, the activities relevant for consumer participation were farm visits, workshops, and events.

Within the *Mexican* legislative framework, the guidelines for organic production recommend the involvement and participation of consumers in the PGS certification committee (Secretaria de Agricultura y Desarrolla Rural 2020). At the Mexican-CSM, consumers were invited to participate in farm visits carried out as part of the guarantee process in Chapingo (MI2) and Tlaxcala (MI3). In Tlaxcala, the aim of permanently including one consumer in the market’s participatory certification committee could not be achieved (MI4). Similarly, in Chapingo consumers only participated in farm visits occasionally (MI2; MI8). In Oaxaca, consumers were not invited to participate in farm visits as the guarantee process was conceived as an internal control of CSM members (MI5). However, special farm visits for consumers were arranged by some members (MI5). This was also the case in Tlaxcala, where most long-standing customers had visited the farms (MI1; Kaufmann and Vogl 2018). The Mexican-CSM in Chapingo regularly held workshops for consumers on market days (MI2). In Tlaxcala, consumers had the opportunity to participate in workshops organized for producers every two months (MI3). At both CSMs, events were held at the marketplace for special occasions (MI2; MI3).

Consumer participation in PGS activities and committees is not defined by *Chile*’s national PGS legislation (Servicio Agrícola y Ganadero 2019), and Chilean-CSM consumers did not have the opportunity to participate in the Tierra Viva PGS certification process (Hruschka et al. 2022; CI2) or in the market’s internal control system. Nonetheless, Chilean-CSM had arranged private farm visits in recent years (CI4) and the consumers surveyed said they visited producers out of personal interest. Consumers were able to participate in cultural events, presentations, and workshops held on market days by an employed cultural event manager (CI4).

In the *Bolivian* context, national legislation requires consumer participation in PGS since the national technical PGS regulation states that consumers with knowledge of organic agriculture must be represented on the PGS evaluation committee (Ministerio de Desarrollo Rural y Tierras 2012). At the Bolivian-CSM, the active participation of consumers in the PGS certification process had been defined as a central objective (BD2). During data collection, a consumer committee at the ECO Feria PGS was identified, however there was no documentation or further information about its origins and functions. Internal protocols from 2012 and representatives from the ECO Feria PGS indicated that consumers had participated in PGS activities, such as the ECO Feria PGS evaluation committee (BD1; BI1). However, during data collection, only private farm visits by consumers were identified. Similar to the consumer committee, participation in the evaluation committee was perceived to be for form’s sake and in order to conform with the Bolivian national legislation on PGS, yet no consumer participation was witnessed by the third author during data collection. PGS meetings were held according to demand at the marketplace of Bolivian-CSM and were open to the general public (BI1). During data collection, no public workshops or events were held by the ECO Feria PGS or the Bolivian-CSM.

#### Consumer participation in PGS and at the CSMs

About one fifth of consumers surveyed had participated in visits to CSM producers’ farms.^2^ Participation was significantly higher at Bolivian-CSM and Chilean-CSM than at Mexican-CSM (Table 3, Appendix A4).

**Table 3:** Consumer participation in visits to producers’ farms at the Mexican case study markets (Mexican-CSM), the Chilean case study market (Chilean-CSM) and the Bolivian case study market (Bolivian-CSM) (n = number)

Variable	Total sample (n = 201, 100% = 199)	Mexican-CSM Chapingo / Tlaxcala / Oaxaca [n = 61 (21/19/21), 100% = n)]	Chilean-CSM Ecoferia de la Reina (n = 82, 100% = n)	Bolivian-CSM ECO Feria Cochabamba (n = 58, 100% = 56)
Participation in farm visits [yes]	19.6%	4.9%	18.3%	37.5%

For Chilean-CSM and Bolivian-CSM, the frequency of attendance at farm visits was also assessed. This question was not put to Mexican-CSM consumers. Consumers at Chilean-CSM and Bolivian-CSM had visited farms between one and 12 times in the year prior to data collection, and 75% had participated once or twice (Table 4).

**Table 4:** Frequency of consumer participation in farm visits at the Chilean case study market (Chilean-CSM) and the Bolivian case study market (Bolivian-CSM) (n = number; x̄ = arithmetic mean; SD = standard deviation; question not asked to Mexican-CSM consumers)

Frequency of participation in farm visits in past year	Total sample Chilean-CSM & Bolivian-CSM (n = 36)	Chilean-CSM Ecoferia de la Reina (n = 15)	Bolivian-CSM ECO Feria Cochabamba (n = 21)
x̄ (SD)	2.61 (2.71)	1.4 (1.06)	3.48 (3.19)
Minimum; maximum; median	1; 12; 1	1; 5; 1	1; 12; 2
Quartiles (25/50/75)	1/1/3.5	1/1/1	1/2/5

Thus, although partially attempted, consumer participation in the PGS certification process was barely observed in the CSMs. In the scientific literature, there is little documentation on consumer-PGS interactions (Kaufmann et al. 2020). Similarly, consumer involvement in AFN has also been found to be difficult to achieve (Cone and Myhre 2000; Forssell and Lankoski 2015; Pole and Gray 2013). Although large numbers of consumers have been indicated as participating in PGS in France and Brazil (Niederle et al. 2020; Rover et al. 2017; Zanasi et al. 2009), in most of the literature consumer participation in PGS activities is indicated to be a challenge (López Cifuentes et al. 2018; Nelson et al. 2016), very low (Bara et al. 2017) or completely absent (Clark and Martínez 2016; Home et al. 2017; Montefrio and Johnson 2019).

Similar to the results presented in AFN literature (Pole and Gray 2013), PGS studies have documented the key role played by consumers and their active involvement in the founding and initial phase of the PGS (Bellante 2017; Chaparro-Africano and Naranjo 2020; López Cifuentes et al. 2018). Yet it would seem that consumer engagement decreases with time (Bellante 2017; Lemeilleur and Sermage 2020; Pole and Gray 2013). These findings are also underlined by the results from the Mexican-CSM in Chapingo and Tlaxcala.

IFOAM recommends to counteract PGS volunteer “burn-out” (IFOAM 2019) by means of financial remuneration and paid employment. Yet the factors limiting consumers’ willingness to participate in PGS activities, such as time requirements (Nelson et al. 2016), lack of perceived expertise to conduct activities such as control visits (Nelson et al. 2016), disinterest (López Cifuentes et al. 2018) or travel distances (Bara et al. 2017), can hardly be counteracted by remuneration. In the context of AFN, Diekmann and Theuvsen (2019) have highlighted the considerable effort consumers believe participation to involve, and its incompatibility with their daily routines. Diekmann and Theuvsen recommended providing consumers with information and clarifying their expectations in order to dismantle barriers to participation and address the compatibility of consumer AFN involvement and their day-to-day-life. In countries that legally recognize PGS as an organic quality assurance system, it could be argued that the legislative framework within which PGS operate provides a powerful tool to predefine consumer-PGS interactions. However, as the data from Bolivia suggest, a legal environment that obliges the involvement of consumers in the PGS and its activities may also lead to a system in which consumers are involved only for form’s sake, without really fostering active participation.

Consumer awareness is a prerequisite for consumers to become involved in AFN (Diekmann and Theuvsen 2019; Sacchi 2018). In this context, information campaigns, workshops and events can be useful tools to foster consumer awareness. Chilean-CSM and Mexican-CSM in Chapingo and Tlaxcala offered workshops and events at the marketplace as additional opportunities for consumers to participate, and about one fifth (19.5%) of the surveyed consumers seized the opportunity to do so (n = 201, 100% = 128). At Mexican-CSM 17.4% (n = 61, 100% = 46) and at Chilean-CSM 20.7% (n = 82, 100% = n) had participated. Chilean-CSM consumers participated between one and twelve times in workshops or events in the year prior to data collection (x̄ = 2.6, median = 2, n = 17). However, as observed at Chilean-CSM, workshops and events were aimed at entertaining market visitors and there was little interest among consumers in information events (CI4). In the Mexican-CSM in Tlaxcala, workshops were primarily arranged for producers and covered very specific topics on organic production (MI3). Only in the Mexican-CSM in Chapingo did consumer workshops address the PGS (MI2); yet those held during the period of data collection covered other topics such as the preparation of traditional meals (MPO3).

By offering participation possibilities in workshops and events, CSMs become places in which to socialize and be part of a community (Dodds et al. 2014; Figueroa-Rodríguez et al. 2019). From an AFN perspective, events contribute to building and strengthening social relations, thus fostering the social embeddedness of economic activities at the CSMs (Maye and Kirwan 2010). Moreover, such workshops and events can attract new consumers and awaken their interest in PGS and CSM. As has been outlined by Diekmann and Theuvsen (2019), “[i]n order to inspire people who have not previously taken part (…) addressing rather hedonic motifs emphasizing pleasure and fulfillment on a personal level (…)” (Diekmann and Theuvsen 2019 p.7) can be a successful strategy.

### PGS relations of trust

While CSM consumers showed low levels of PGS awareness and active participation in the PGS initiative, when asked to indicate their level of trust in products sold as organic at the CSM actually being organic, on a six-level ordinal scale from zero (no trust) to six (complete trust), consumers generally reported high levels of trust, with a median of four (Table 5).

**Table 5:** Consumers’ trust in organic product quality at the Mexican case study markets (Mexican-CSM), the Chilean case study market (Chilean-CSM) and the Bolivian case study market (Bolivian-CSM) (six-point ordinal scale, 0 = no trust, 6 = complete trust; n = number)

Trust that organic products sold at the case study market are organic		Total sample (n = 201, 100% = 199)	Mexican-CSM Chapingo / Tlaxcala / Oaxaca [n = 61 (21/19/21), 100% = 60]	Chilean-CSM Ecoferia de la Reina (n = 82, 100% = n)	Bolivian-CSM ECO Feria Cochabamba (n = 58, 100% = 57)
Survey response option	None / very low / low	3%	3.3%	2.4%	3.5%
	Regular	24.6%	25%	7.3%	49.1%
	High / very high / complete	72.4%	71.7%	90.2%	47.4%

In contrast to Mexican-CSM and especially Chilean-CSM consumers, who showed the highest level of trust (Table 5), the results characterized Bolivian-CSM consumers as not only having a very low awareness of the PGS certification system, but also an overall low level of trust in the organic quality of Bolivian-CSM products. Consumers at Bolivian-CSM showed significantly lower trust in the integrity of the organic products (Appendix A4).

These findings from Bolivian-CSM resonate with Chambilla (2014), who indicates that Bolivian organic consumers generally lack trust in organic labels and products. The Bolivian organic scene has been characterized by domestic debates differentiating ‘organic’ from ‘agro-ecological’ production, and the connotation that organic certification is the weaker of the two systems (Loconto 2016). Home et al. (2017) indicate that parallel certification systems at PGS markets, such as those found at Bolivian-CSM, may prompt doubt among consumers about organic product quality. Mislabeling and product misrepresentation may also induce doubt and stop consumers from purchasing organic produce (Yiridoe et al. 2005). The results from Bolivian-CSM hinted at a similar finding.

To obtain greater insight into consumer trust, consumers’ reasons for trust at Chilean-CSM and Bolivian-CSM were assessed. Results revealed organic producers and CSMs themselves to be the most important factors, and further underlined the lack of trust and information among Bolivian-CSM consumers.

Consumers of Chilean-CSM primarily indicated the producer (48%), followed by certification (22%) and the market (21%) (n = 82, 100% = n) as reasons for their trust. Of the 22% of Chilean-CSM consumers who indicated certification as a reason for trust in the organic quality, 89% did not know about PGS and 61% did not recognize the names of the PGS initiatives operating at the CSM. Consumers’ trust at Bolivian-CSM was primarily associated with product characteristics and benefits (30%) and the producer (14%). Bolivian-CSM consumers indicated a general lack of knowledge (21%) and/or distrust (16%) (n = 56, 100% = n). Certification was not mentioned at all (0%) by Bolivian-CSM consumers (open question, multiple responses possible).

**Fig. 1:**
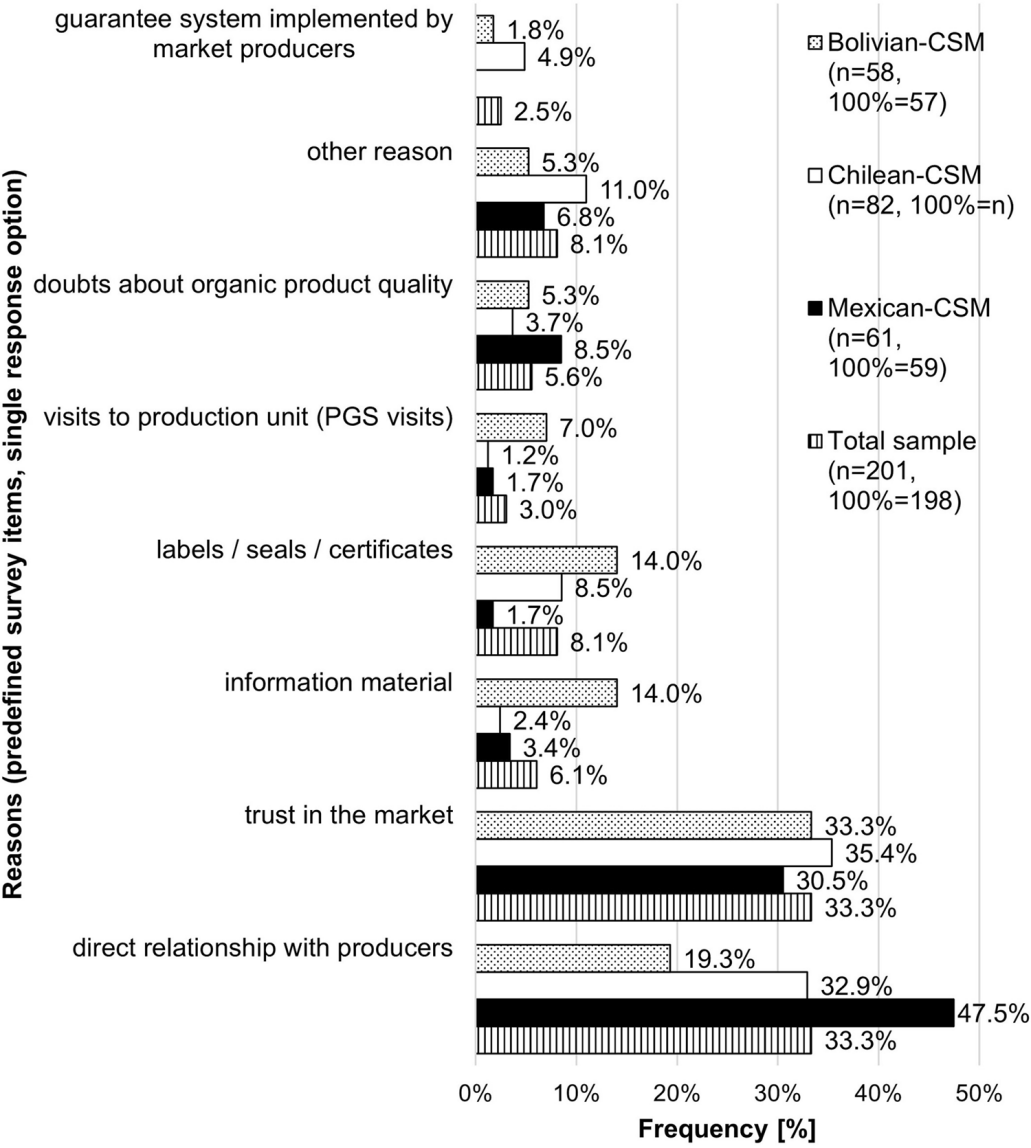
Reasons given by consumers at Mexican-CSM, Chilean-CSM and Bolivian-CSM for knowing that products sold at the CSM were organic (n = 201) (pre-defined item set, single response option; other reason: reasons mentioned by consumers additionally to pre-defined item set; item “guarantee system implemented by market producers” not included at Mexican-CSM)

The reasons most frequently given for knowing that organic products sold at the CSMs were organic were the direct relationship with producers (33.3%) and trust in the market (33.3%) (n = 201, 100% = 198) (Fig. 1). Some consumers expressed doubt about the organic quality of products sold as organic at their respective CSM.

Consequently, these results supported those of Sacchi (2018) who found organic certification not to be a main motivation for consumers to purchase AFN products. Results furthermore resonate with van Truong et al. (2022) who report that consumers considered trust in organic producers more important than certification. Furthermore, the results support the argument that consumer trust comes from an interplay between personal and institutional trust (Kriege-Steffen et al. 2010), where the absence of the one requires even stronger pronouncement of the other and vice versa (van Truong et al. 2022). However, while there was personal trust in CSM members, institutional trust was associated with the market rather than the PGS certification system. The survey design with predefined survey items that were not further specified did not allow for a more profound interpretation of the specific reasons behind responses such as “trust in the market”. The results from Chilean-CSM stood out, as one fifth of consumers mentioned organic certification as one of the sources of trust yet were largely unaware of the PGS. These results hint at a broader, rather superficial awareness among Chilean consumers of organic certification requirements (Rodrigues et al. 2016), a general trust placed in organic certification as an institution (Kriege-Steffen et al. 2010), and the certification’s value as a “good reason” for trusting and purchasing organic products (Zagata and Lostak 2012). In accordance with Eden et al. (2008) and Zagata and Lostak (2012), awareness of the certification system is only one of the conditions contributing to its success, as uninformed consumers “do not have to know very much about organic agriculture” (Zagata and Lostak 2012, p.482) to make purchasing decisions in favor of organic products, but “need to know enough” (Zagata and Lostak 2012, p.482) to be able to establish faith in the products (Zagata and Lostak 2012) or in a particular label (Nilsson et al. 2004; Meixner and Haas 2016). It appeared that consumers did not require more specific knowledge of the PGS to establish a very high level of trust in it. However, due to their very high level of trust, consumers may not have considered more profound knowledge about PGS necessary (Thorsøe and Kjeldsen 2016).

The results furthermore supported a thesis of a strong emotional aspect to trust in organic marketplaces (Meijboom et al. 2006) and observations of PGS and their markets as being characterized by a strong consumer-producer relationship (Carlón 2015) and as an environment in which consumers are able to socialize (Kumpuniemi 2019). CSM consumers’ trust in organic product integrity may thus be determined by these factors rather than by their awareness and knowledge about certification systems in place at CSMs. However, the high levels of consumer trust can be regarded as a pre-condition for engagement and a sound basis for further interactions with CSM producers (Martindale 2020; Mount 2012; Thorsøe and Kjeldsen 2016).

Chilean-CSM, similarly to the Mexican-CSM in Chapingo and Tlaxcala, was characterized by a long history of consumer attendance, high regularity of market attendance and consumers voluntarily visiting producer farms, indicating a willingness to invest time and resources and an interest in interacting with producers beyond product purchase.

**Fig. 2:**
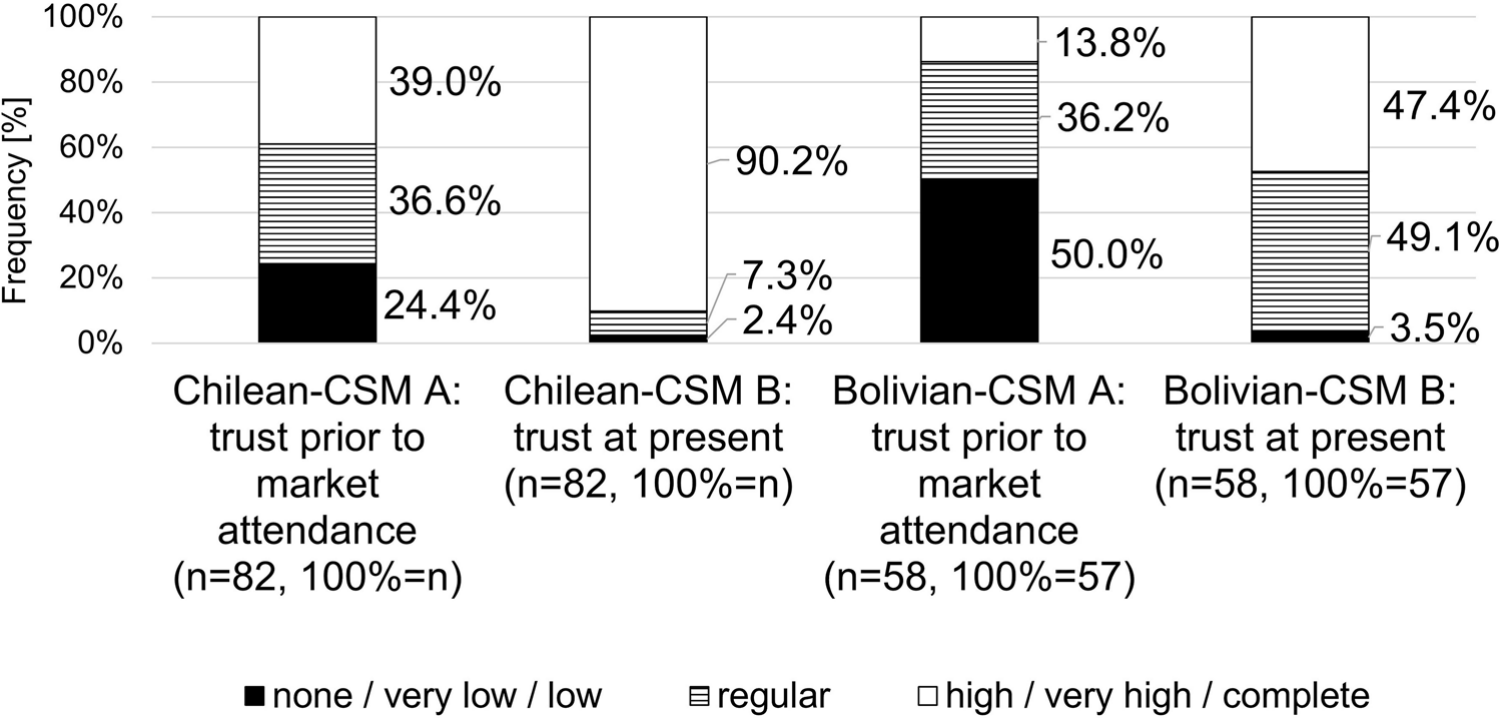
Consumers’ trust in organic product quality prior to case study market attendance (**A**) and at the time of data collection (**B**) at the Chilean case study market (Chilean-CSM) and Bolivian case study market (Bolivian-CSM) (n = 140; six-point ordinal scale, 0 = no trust, 6 = complete trust; question not put to Mexican-CSM consumers)

To explore a potential relationship between consumer market attendance and their trust, consumers at Chilean-CSM and Bolivian-CSM were asked to indicate their level of trust in organic CSM products before they started going to the respective CSM. Consumers at both markets indicated lower trust in the period before they started going to the market (Fig. 2).

Trust prior to CSM attendance was significantly lower for consumers of Bolivian-CSM than those of Chilean-CSM (Appendix A4), and consumers at both CSMs showed significantly greater trust at the time of data collection than before they started going to their market (Appendix A4). This perceived change in consumer trust witnessed at both CSMs might be partially related to the interaction with producers at the market (Chen et al. 2019; Taufique et al. 2019). Yet as consumers were asked to evaluate their trust prior to CSM attendance retrospectively, we cannot exclude potential social desirability effects (Bernard 2006) in the results.

Consumer trust in organic products has been closely related to the visibility and knowledge of the organic logo at the marketplace (Janssen and Hamm 2012; Zagata and Lostak 2012). Moreover, institutional trust has been attributed to enhanced personal trust (Goodman and DuPuis 2002; Renting et al. 2012), while rendering consumer trust more independent from personal trust in individual producers (Diekmann and Theuvsen 2019) and more resilient to possible marketplace inconsistencies (Drescher et al. 2012). Although Chilean-CSM, the Mexican-CSM in Chapingo and Tlaxcala, and to a lesser extent Bolivian-CSM stressed the display of the organic quality of the products, the absence of information on the specifics of the PGS certification system may lessen consumer trust and prevent consumers from obtaining the information needed to deal with possible visible inconsistencies at the marketplace (Zagata and Lostak 2012). In the case of Bolivian-CSM in particular, clear product labeling and communication for consumers could minimize doubt and distrust, balance information asymmetries, and positively direct Bolivian-CSM consumers’ purchasing decisions in favor of organic Bolivian-CSM products (Rodrigues et al. 2016). In this context, the consumer committee’s origins, functions and effects at Bolivian-CSM are still unclear.

Improved consumer information about the PGS and the certification process would also convey the effort made by producers to meet organic standards, thus potentially boosting consumers’ willingness to pay price premiums (Hoogland et al. 2007) and securing sustainable demand (Lunde 2018 cit. Xie et al. 2015). This could be specifically relevant in the CSMs as consumers attached great importance to organic certification systems. Despite their limited PGS awareness and the low relevance of labels, seals and certificates for knowing that CSM products were organic (Fig. 1), consumers attributed great importance to having some certification system in place to reinforce their trust in organic product quality, with a median of four on a scale from zero (no importance) to five (very high importance) (Table 6). A certification system was significantly less important for consumers at Bolivian-CSM than for consumers at Chilean-CSM and Mexican-CSM (Appendix A4).

**Table 6:** Importance of a certification system for reinforcing consumer trust at the Mexican-CSM, the Chilean-CSM and the Bolivian-CSM (n = number)

Importance of certification system for reinforcing consumer trust		Total sample (n = 201, 100% = n)	Mexican-CSM Chapingo / Tlaxcala / Oaxaca [n = 61 (21/19/21), 100% = n]	Chilean-CSM Ecoferia de la Reina (n = 82, 100% = n)	Bolivian-CSM ECO Feria Cochabamba (n = 58, 100% = n)
Survey response option	No / very little / little importance	9.5%	3.3%	4.9%	22.4%
	Moderate importance	17.9%	11.5%	18.3%	24.1%
	High importance	32.3%	37.7%	32.9%	25.9%
	Very high importance	40.3%	47.5%	43.9%	27.6%

To ensure that organic certification and the respective seals and labels effectively counteract consumer-producer knowledge asymmetries regarding organic product qualities, organic food product outlets specifically require knowledge exchange, communication and increased trust (Janssen and Hamm 2012). Farmers’ markets are no exception, even though producer–consumer information asymmetries may be overcome more easily by means of direct producer–consumer interactions. While the social embeddedness attributed to face-to-face interactions between consumers and producers has been indicated as contributing to a more integrated community (Hinrichs 2000), inducing sympathy and building trust (Chen et al. 2019; Kriege-Steffen et al. 2010), the elements of marketness and instrumentalism are not completely neutralized, and product price as well as consumers’ willingness to pay it still remain relevant (Hinrichs 2000). Furthermore, personal producer–consumer relations may not be enough to safeguard consumer trust in the long-term (Granovetter 1985). Therefore, personal assurance through interpersonal ties and personal trust-building based on face-to-face encounters on the one hand, and knowledge of the certification schemes provided by trustworthy sources that facilitate the formation of institutional trust on the other, complement each other ideally for establishing consumer trust in PGS and their markets (Chen et al. 2019; Thamchaisophis 2021; Thorsøe and Kjeldsen 2016).

## Conclusions and outlook

PGS are viewed as an increasingly interesting option for smallholder organic certification, offering a way to guarantee organic product quality to consumers and build trust among stakeholders in the food chain. Furthermore, PGS are considered to potentially increase consumer awareness and the consumption of organic products and provide a platform for valuable producer–consumer interactions.

CSM consumers were characterized by a long history and high frequency of market attendance and high levels of trust in organic CSM products, but low awareness of PGS and little participation in PGS activities. Similarly to results presented by Kato and McKinney (2015), it cannot be excluded that the lack of consumer participation is directly linked to lack of awareness of the PGS. CSMs showed themselves to be places of socialization (Kumpuniemi 2019), where economic market activities are socially embedded in strong producer–consumer relationships (Carson et al. 2016; Maye and Kirwan 2010). Consumers relied primarily on these direct relationships and the market itself as a source of trust. With the exception of Chilean-CSM, PGS certification was not an important reason for trust and was not considered a major factor for identifying organic products at the marketplace. Nevertheless, consumers attached great importance to the existence of a certification system to formally support their trust in organic CSM products.

The results also showed differences between the five CSMs studied and underlined how the structure and intent of the individual PGS initiatives predefine the extent of the potential for participation (IFOAM 2019). Overall, consumers showed interest and a willingness to interact with CSM producers outside the market and the PGS.

By purchasing CSM products, consumers assume an important role in the CSM and the PGS. However, based on the survey results, CSM consumers did not yet assume the active, catalyst role of civic engagement that they are said to adopt (Goodman and DuPuis 2002; Renting et al. 2012) and showed to do so in AFN (Sacchi et al. 2022). As exemplified by the Bolivian- and Chilean-CSM, PGS markets still have potential to further differentiate themselves from conventional farmers’ markets. Further research on CSM consumers, particularly those of Bolivian-CSM, is desirable to deepen the initial insight offered by this study. In this context, it would be of interest in future studies to identify whether the distrust in organic quality expressed by Bolivian-CSM consumers is culturally predisposed or if it is driven by the information asymmetry of the certification systems and the presence of vendors selling non-certified organic produce.

It was not the intention of this study to explore each of the presented concepts of awareness, participation, trust and social embeddedness to their full extent. Focusing on embeddedness as a singular area when analyzing economic interactions has been critiqued as being inconclusive (Hinrichs 2000; Winter 2003), leading to an oversimplification and an “overly sentimental view” (Sage 2003) of interactions at the marketplace if aspects of marketness and instrumentalism are not considered (Hinrichs 2000; Sage 2003). While acknowledging these arguments, the approach chosen for this study nevertheless allowed a broadening of the discussion on producer–consumer economic interactions and their social embeddedness in Latin American AFN, specifically in PGS. It is yet to be seen to what degree these findings continue to be valid in the wake of local political and economic developments and changes in local and global organic market dynamics in the past years.

To draw a more comprehensive picture of PGS consumers and conduct an in-depth assessment of the potential for further developing PGS and their markets, future research may consider marketness and instrumentalism (Hinrichs 2000), and explore the relevance of organic product attributes for consumers’ purchasing decisions (Higgins et al. 2008).

If consumer involvement in PGS is seen as key to guaranteeing the success of PGS initiatives (Clark and Martínez 2016; Home et al. 2017) and their organic markets, the development of strategies to increase consumer participation is crucial for the CSMs studied. Consumer participation could foster the promoted benefits of PGS, such as shared learning and responsibility, further trust-building, and the creation of social networks (IFOAM 2018, 2019). By joining PGS farm visits, consumers could verify organic production processes and cross-check their idea of organic farming with agricultural realities directly on the farm, increasing transparency and improving their understanding of organic production processes (Diekmann and Theuvsen 2019). This could result in a better informed, more aware and ultimately empowered community of PGS and CSM consumers (Forssell and Lankoski 2015). Considering the high levels of consumer trust illustrated by the empirical data, CSMs are in a good starting position to promote further the active participation of consumers in PGS and CSM activities (Martindale 2020; Thorsøe and Kjeldsen 2016).

Our results compare CSMs in three different historical, political, social and economic diverse countries. We acknowledge the importance of considering these dimensions when interpreting the results. Amongst the influence of possible interviewer effects, different data collection timeframes and difficulties in the comparability of the three countries, the finally detected unreliability of consumers’ answers on their economic status represents a central limitation of this study. Comparing survey respondents’ educational level to OECD and national country data indicates that university graduates were strongly overrepresented in the survey sample as compared to the three countries’ overall population (INE Bolivia 2018; OECD 2021). Educational levels have shown to correlate with income levels in Chile (INE Chile 2018) and Gaitán-Cremaschi et al. (2020) classified consumers of PGS products in Chile among medium to high socio-economic income groups, as did key informants at the Chilean-CSM and two Mexican-CSM. However, without considering economic or social factors in our results, we are unable to thoroughly indicate if the CSMs promote inclusivity and food sovereignty or rather represent the segregation of a specific, academic, economic well-situated consumer strata, from conventional markets in Latin America. We recommend future studies to address these limitations. Either way, the potential of CSMs and likeminded initiatives to challenge the conventional food regime should not be overlooked (Sacchi 2018).

Based on the data presented in this paper, the potential of workshops and events for informing and educating consumers about the PGS and organic production processes, as a pre-condition for increased consumer involvement, does not yet appear to be exploited fully by the CSMs (Carson et al. 2016; Diekmann and Theuvsen 2019). Beyond enhanced consumer participation, raising consumer awareness and knowledge of PGS and the certification system could further facilitate consumers’ institutional trust in the certification system, making this a worthwhile activity for boosting consumer trust (Kriege-Steffen et al. 2010; Meixner and Haas 2016). Such efforts are imperative for securing demand for PGS-certified products and fostering PGS markets as places of consumer-producer interaction, knowledge exchange, and trust building (Batte et al. 2007; Giovannucci and Ponte 2005; Higgins et al. 2008; Janssen and Hamm 2012; Maye and Kirwan 2010). Upholding additional verification processes, such as internal market controls, and communicating them to consumers could further contribute to shifting trust away from a primarily producer/consumer-focused relationship towards a rational evidence-based approach.

Consequently, the following suggestions are made to facilitate consumer PGS awareness and participation and further develop PGS and CSMs:

1. Increase consumer awareness of PGS through locally adapted, easily understandable messages, explicitly use workshops and events at the marketplace to communicate PGS to consumers, and inform consumers about organic production and verification processes
2. Use close direct producer–consumer relationships to spread information more effectively about the PGS and organic farming among consumers
3. Apply consistent labeling mechanisms for organic CSM products and more effective control mechanisms at the marketplace to avoid inconsistencies in product labeling, reduce doubt, safeguard CSM integrity, and support consumer trust
4. Foster an open, structured dialogue with consumers that explicitly addresses their interests, expectations, concerns and barriers regarding PGS participation, and explores possibilities of consumer participation in parallel with their day-to-day routines.

## Appendix 1: Data sources

See Table 7.

**Table 7:** Details on data sources collected in Mexico, Chile and Bolivia (n.d.: no date available; Mexican-CSM = Mexican case study markets; Chilean-CSM = Chilean case study market; Bolivian-CSM = Bolivian case study market)

Code	Country	Material	Source	Organization	Date
BI1	Bolivia	Semi-structured interview	PGS representative	Asociación SPG ECO Feria (Bolivian-CSM)	17.07.2019
BI2	Bolivia	Semi-structured interview	PGS representative	AGRECOLANDES	14.06.2019
BD1	Bolivia	Document analysis	Assembly protocol	Asociación SPG ECO Feria (Bolivian-CSM)	27.06.2012
BD2	Bolivia	Document analysis	Internal regulation on structure and functioning of the PGS ECO Feria	Asociación SPG ECO Feria (Bolivian-CSM)	n.d
BPO1	Bolivia	Participant observation	Presentation “Taller RA 017/2012 Resolución ministerial. Manual de procedimientos para el registro y autorización de los SPGs en el SNCPE (Sistema Nacional de Control de la Producción Ecológica).”	Municipalidad de Sacaba	11.07.2019
CI1	Chile	Semi-structured interview	Market representative	Ecoferia de la Reina (Chilean-CSM)	30.10.2019
CI2	Chile	Semi-structured interview	PGS representative	Tierra Viva Asociación Gremial	04.06.2019
CI3	Chile	Semi-structured interview	PGS representative	Tierra Viva Asociación Gremial	29.06.2019
CI4	Chile	Semi-structured interview	Market representative	Ecoferia de la Reina (Chilean-CSM)	21.08.2019
CI5	Chile	Semi-structured interview	Market representative	Ecoferia de la Reina (Chilean-CSM)	12.06.2019
CI6	Chile	Semi-structured interview	Market representative	Ecoferia de la Reina (Chilean-CSM)	21.06.2019
MI1	Mexico	Semi-structured interview	Member of the certification committee	Tianguis Alternativo Tlaxcala (Mexican-CSM)	11.12.2015
MI2	Mexico	Semi-structured interview	Market coordinator	Tianguis Orgánico Chapingo, Texcoco (Mexican-CSM)	05.12.2015
MI3	Mexico	Semi-structured interview	Market coordinator	Tianguis Alternativo Tlaxcala (Mexican-CSM)	11.12.2015
MI4	Mexico	Semi-structured interview	Member of the certification committee	Tianguis Alternativo Tlaxcala (Mexican-CSM)	04.12.2015
MI5	Mexico	Semi-structured interview	Market coordinator	Tianguis Alternativo el Pochote Xochimilco, Oaxaca de Juárez (Mexican-CSM)	28.11.2015
MPO1	Mexico	Participant observation	Participant observation protocol	Tianguis Orgánico Chapingo, Texcoco (Mexican-CSM)	01.10.2015
MPO2	Mexico	Participant observation	Participant observation protocol	Mercado Alternativo Tlaxcala (Mexican-CSM)	13.11.2015
MPO3	Mexico	Participant observation	Participant observation protocol	Tianguis Orgánico Chapingo, Texcoco (Mexican-CSM)	09.2015–03.2016
MPO4	Mexico	Participant observation	Participant observation protocol	Mercado Alternativo Tlaxcala (Mexican-CSM)	10.2015–03.2016
MPO5	Mexico	Participant observation	Participant observation protocol	Tianguis Alternativo el Pochote Xochimilco (Mexican-CSM)	11.2015–01.2016
MD1	Mexico	Document analysis	Internal regulation on participatory certification	Tianguis Orgánico Chapingo, Texcoco (Mexican-CSM)	01.2016
MD2	Mexico	Document analysis	Presentation: “9 Años de Experiencia Mercado Alternativo”	Mercado Alternativo Tlaxcala (Mexican-CSM)	n.d

## Appendix 2: Sample description

See Table 8.

**Table 8:** Sample description: demographics of consumers surveyed at the Mexican case study markets (Mexican-CSM), the Chilean case study market (Chilean-CSM) and the Bolivian case study market (Bolivian-CSM) (n = number; x̄ = arithmetic mean; SD = standard deviation; Min. = minimum; max. = maximum)

Variable		Total sample	Mexican-CSM	Chilean-CSM	Bolivian-CSM
		(n = 201)	Chapingo / Tlaxcala / Oaxaca [n = 61 (21/19/21)]	Ecoferia de la Reina (n = 82)	ECO Feria Cochabamba (n = 58)
Sex (100% = n)	Female	59.2%	47.5%	68.3%	58.6%
	Male	39.8%	52.5%	31.7%	37.9%
	Other	1%	0%	0%	3.4%
Age (years)	n valid	200	61	82	57
	x̄ (SD)	42.55 (14.75)	45.44 (15.75)	44.72 (13.48)	36.33 (13.75)
	Min.; max	19; 77	21; 74	21; 77	19; 75
Education (100% = n)	Primary school	1%	3.3%	0%	0%
	Secondary school or high school	17.9%	21.3%	13.4%	20.7%
	University	78.1%	70.5%	86.6%	74.1%
	Other	3%	3%	0%	5.2%
Travel time to marketplace (minutes)	n valid	197	60	82	55
	x̄ (SD)	21.90 (21.01)	22.03 (16.12)	19.16 (13.17)	25.88 (32.10)
	Min.; max	3; 240	3; 75	5; 80	3.5; 240
	Quartiles (25/50/75)	10/20/30	10/15/30	10/15/25	10/20/30

## Appendix 3: Length of consumer market attendance

See Table 9.

**Table 9:** Time of consumer market attendance at the Mexican case study markets (Mexican-CSM), the Chilean case study market (Chilean-CSM) and the Bolivian case study market (Bolivian-CSM) (n = number; x̄ = arithmetic mean; SD = standard deviation; Min. = minimum; max. = maximum)

Variable	Statistics	Total sample (n = 201, 100% = n)	Mexican-CSM Chapingo / Tlaxcala / Oaxaca [n = 61 (21/19/21), 100% = n]	Chilean-CSM Ecoferia de la Reina (n = 82, 100% = n)	Bolivian-CSM ECO Feria Cochabamba (n = 58, 100% = n)
Length of market attendance (years)	n valid	201	61	82	58
	x̄ (SD)	3.24 (3.32)	3.72 (3.65)	3.72 (3.06)	2.08 (3.08)
	Min.; max.; mode	0; 15; 2	0; 12; 2	0.1; 14; 5	0; 15; 0.2, 2
	Quartiles (25/50/75)	0.5/2/5	1/2/6	1/3/5	0.2/1/3
	Accumulative percentage				
	> 1 year	61.2%	63.9%	72%	43.1%
	> 2 years	43.8%	44.3%	56.1%	25.9%
	> 3 years	34.8%	36.1%	47.6%	15.5%
	> 4 years	29.9%	34.4%	37.8%	13.8%
	> 5 years	20.4%	27.9%	22%	10.3%

## Appendix 4: Differences between CSM

See Table 10.

**Table 10:** Overview of statistically significant differences between markets: for pairwise comparison between markets, only pairs with statistically significant results displayed (n.a. = survey item not asked; – = market not included in statistical test; n = number; χ2 = Chi-square; p = p-value)

Variable	Test and test statistics	Mexican-CSM Chapingo / Tlaxcala / Oaxaca (n = 21/19/21)	Chilean-CSM Ecoferia de la Reina (n = 82)	Bolivian-CSM ECO Feria Cochabamba (n = 58)
Length of market attendance [years]	Kruskal–Wallis H test, χ2 = 17.957 p = 0.000***, n = 201	Mean rank = 107.59	Mean rank = 115.02	Mean rank = 74.25
	Mann–Whitney U test, U = 1207.5, Z = -3.00, p = 0.000***, n = 119	Mean rank = 69.2 Sum of ranks = 4221.5	–	Mean rank = 50.32 Sum of ranks = 2918.5
	Mann–Whitney U test, U = 1388, Z = -4.203, exact p = 0.000***, n = 140	–	Mean rank = 82.57 Sum of ranks = 6771	Mean rank = 53.43 Sum of ranks = 3099
Frequency of market attendance (times per year)	Kruskal–Wallis H test, χ2 = 6.856 p = 0.032*, n = 194	Mean rank = 93.27	Mean rank = 108.74	Mean rank = 85.41
	Mann–Whitney U test, U = 1768, Z = -2.542, exact p = 0.011*, n = 139	–	Mean rank = 76.94 Sum of ranks = 6309	Mean rank = 60.02 Sum of ranks = 3421
Heard about PGS in general	χ^2^-test, χ^2^ = 4.085, p = 0.043*, n = 135, phi = -0.174, p = 0.043*	–	14.6% (n = 82, 100% = n)	3.8% (n = 58, 100% = 53)
	χ^2^-test, χ^2^ = 9.685, p = 0.002**, n = 114, phi = -0.291, p = 0.002**	24.6% (n = 61, 100% = n)	–	3.8% (n = 58, 100% = 53)
Heard about PGS initiative	χ^2^-test, χ^2^ = 6.423, p = 0.011*, n = 140, phi = -0.214, p = 0.011*	n.a	39% (n = 82, 100% = n)	19% (n = 58, 100% = n)
Participation in farm visits	χ^2^-test, χ^2^ = 5.687, p = 0.017*, n = 143, phi = 0.199, p = 0.017*	4.9% (n = 61,100% = n)	18.3% (n = 82, 100% = n)	–
	χ^2^-test, χ^2^ = 19.009, p = 0.000***, n = 117, phi = 0.403, p = 0.000***	4.9% (n = 61,100% = n)	–	37.5% (n = 58, 100% = 56)
Frequency of participation in farm visits per year	Mann–Whitney U test, U = 78, Z = -2.780, exact p = 0.005**, n = 36	n.a	Mean rank = 13.20 Sum of ranks = 198	Mean rank = 22.29 Sum of ranks = 468
Trust that organic products sold at the case study market are organic	Kruskal–Wallis H test, χ2 = 48.56 p = 0.000***, n = 201	Mean rank = 87.49	Mean rank = 131.66	Mean rank = 67.62
	Mann–Whitney U test, U = 899, Z = -6.346, exact p = 0.000***, n = 139; Fisher’s exact test, χ2 = 33.131, exact p = 0.000***, n = 139, Cramer’s V = 0.485, exact p = 0.000***	–	Median = 5 Mean rank = 87.54 Sum of ranks = 7178	Median = 3 Mean rank = 44.77 Sum of ranks = 2552
	Mann–Whitney U test, U = 1302.5, Z = -2.350, p = 0.019*, n = 117; Fisher’s exact test, χ2 = 7.598, exact p = 0.012*, n = 117, Cramer’s V = 0.253, exact p = 0.017*	Median = 4 Mean rank = 65.79 Sum of ranks = 3947.5 =	–	Median = 3 Mean rank = 51.85 Sum of rank = 2955.5
	Mann–Whitney U test, U = 1302, Z = -4.949, exact p = 0.000***, n = 142; Fisher’s exact test, χ2 = 8.828, exact p = 0.006**, n = 142, Cramer’s V = 0.250, exact p = 0.006**	Median = 4 Mean rank = 52.2 Sum of ranks = 3132	Median = 5 Mean rank = 85.62 Sum of ranks = 7021	–
Importance of certification system	Mann–Whitney U test, U = 1691, Z = -3.042, exact p = 0.002**, n = 140	–	Mean rank = 78.88 Sum of ranks = 6468	Mean rank = 58.66 Sum of ranks = 3402
	Mann–Whitney U test, U = 1131.5, Z = -3.558, p = 0.000***, n = 119	Mean rank = 70.45 Sum of ranks = 4297.5	–	Mean rank = 49.01 Sum of ranks = 2842.5
Trust prior to case study market attendance	Mann–Whitney U test, U = 1581, Z = -3.475, exact p = 0.000***, n = 140; χ2-test, χ2 = 13.937, exact p = 0.001**; n = 140	n.a	Mean rank = 80.22 Sum of ranks = 6578	Mean rank = 56.76 Sum of ranks = 3292
Trust at the time of data collection & trust prior to case study market attendance	Wilcoxon Signed Rank test, Z = -8.021, p = 0.000***, n = 139	n.a	Z = -6.471, p = 0.000***, n = 82	Z = -4.770, p = 0.000***, n = 57

## Appendix 5: Frequency of consumer market attendance

See Table 11.

**Table 11:** Frequency of consumer market attendance at the Mexican case study markets (Mexican-CSM), the Chilean case study market (Chilean-CSM) and the Bolivian case study market (Bolivian-CSM) (n = number; x̄ = arithmetic mean; SD = standard deviation; min. = minimum; max. = maximum; χ2 = Chi-square; p = p-value)

Variable	Statistics	Total sample (n = 201, 100% = 194)	Mexican-CSM Chapingo / Tlaxcala / Oaxaca [n = 61 (21/19/21), 100% = 55)]	Chilean-CSM Ecoferia de la Reina (n = 82, 100% = n)	Bolivian-CSM ECO Feria Cochabamba (n = 58, 100% = 57)
Frequency of case study market attendance	Descriptive statistics [times per year]				
	n valid	194	55	82	57
	x̄ (SD)	35.73 (17.76)	33.31 (15.88)	39.44 (17.66)	32.74 (18.9)
	Min.; max.; mode	2; 99; 48	2; 48; 48	6; 96; 48	12; 99; 48
	Quartiles (25/50/75)	24/36/48	12/36/48	24/48/48	18/24/48
	Accumulative percentage				
	> 1x/month	80.4%	74.5%	87.3%	75.4%
	> 2x/month	59.8%	58.2%	68.3%	49.1%
	1x/week	41.8%	47.3%	46.3%	29.8%

## Abbreviations

AFN: Alternative Food Networks
CSA: Community Supported Agriculture
CSM: Case Study Market
PGS: Participatory Guarantee Systems
TPC: Third-Party Certification

## Footnotes

1 According to the Global Organic Trade Guide 2016–2022 e.g. Brazil: 15%, Chile: 18%, Mexico: 23%, Peru: 26%; Germany: 11%, US: 6% (Global Organic Trade Guide 2022a; 2022b; 2022c; 2022d; 2022e; 2022f).

2 At -Chilean-CSM and Bolivian-CSM, the survey asked consumers whether they had visited producers’ farms to find out about their production, while at Mexican-CSM the question was specifically directed at farm visits as part of the participatory certification process (“*visitas de acompañamiento”*).
